# Coeliac disease, mucosal change and IEL: doing what counts the best 

**Published:** 2016

**Authors:** Michael N. Marsh

**Affiliations:** *Wolfson College, University of Oxford, UK*

There has been a flurry of activity in GHFBB arising from my classification (1), and I am flattered that the editors have now asked me for an overview. Readers should note that the flurry originated from a recent paper (2) in which we revealed the redundancy of Oberhuber's re-classification of the Marsh III coeliac lesion. First, we indicated that there is no structural basis for his assertion. Second, there is a need to recognise that his subdivisions have never offered useful contributions either to diagnosis, response to treatment, or longer-term outlook. So why use them? And, for those who had the initiative to enquire, no correspondence exists between Oberhuber's subdivisions (despite their technically poor quality) and those of others (3), despite both being offered as supportive guides for histopathologists. That observation emphasises the subjectivity of the exercise. But that revelation hardly surprises, since no structural criteria were ever produced to guarantee reproducibly standard sub-classifications. That is the impasse, but one which is easily by-passed.

 The flurry of editorials and correspondence gave rise to a completely different focus, unfortunately, from that intended in our paper. That outcome asked an entirely different question: which classification is the best? No answer emerged. What to me matters is that histopathologists (most likely through pattern recognition) can spot the infiltrated lesions of Marsh I and Marsh II, as well as a flat lesion (Marsh III) in comparison with "normal" (Marsh O). For university-educated people, is that too much for them to hold in their brains? What real value obtains by reducing those simple precepts? 

 Let me elucidate.

 First, however, some preliminaries need to be aired as foundational for this review. My paper in *Gastroenterology* (1992) was an attempt to entice the pursuit of "coeliac disease" out of the doldrums of its c1950-oriented thinking and practice, and catapult it into the molecular world of the 21^st^ Century (1). That involved bringing together all the known sequences of the gliadins and cognate proteins as a first step towards predicting likely immunogenic epitopes, and secondly, considering possible polymorphisms in the HLA genes of those sensitised to gluten. Next, I suggested that the coeliac-associated lymphomas should be tabulated and classified. We now have two internationally-acclaimed units (at least) in Paris and Amsterdam which have achieved that aim with distinction. Fourthly, I had also realised, as hinted at sporadically in the literature, that there was a spectrum of mucosal changes preceding the flat mucosa which had reigned supreme over the previous 40 years. 

 During that long period no-one, it seems, had ever asked how a normal, villus-bearing mucosa could become flat. And concurrently, that problem was seemingly intensified after the publication of what seemed to be the most "non-coeliac-like" infiltrated villi exemplified by dermatitis herpetiformis (4). I never imagined at that time that I would be the first to amalgamate all these lesions into a credible procession of changes.

 It was therefore of greater surprise to me – because that had never entered my mind either - that the original classification would become the cornerstone of diagnostic usage, which still persists today. In fact, it could not be any different, because that structural curriculum and its simplified pathogenetic underpinnings, fully accounts for the mucosal responses to gluten ingestion seen in genetically-predisposed individuals. 

 But the diagram illustrating the sequence of changes (1) was a *structural* account. It seems, as Corazza and Villanacci very rightly pointed out some years ago (5), that the Marsh II lesion is the least commonly observed. Thus, recognition of *temporal* rates of mucosal change have additionally to be borne in mind as part of the evolving mucosal landscape. Of this we have scant knowledge, while Oberhuber's venture into this field has not brought any new incisive insights. Thus to return, any attempt to excise or elide Marsh II, however kindly a gesture towards diagnostic histopathologists, loses an important aspect of the underlying mechanism(s) that initiate, perpetuate, and progress these changes. Moreover, we need far more knowledge of these many contributory factors than is currently understood.

 Nevertheless, I think Walker (7) is quite right in emphasising that the diagnosis comes at the bedside, and not down a microscope. I was never a board- or College-certified pathologist, but a physician with a very special interest, my training having been acquired at BU Medical Center, Boston (1970-1), through the Rubin-Trier link. Despite that, I could not (legally) report on biopsies processed in my research lab. As a corollary, it is most important that histopathologists recognise that Marsh I and II lesions belong to a diagnostic set now characterised within the scope of Rostami's 'Microscopic Enteropathy' (8). 

 Therefore, it should be recognised and understood that there is no histological state evoking the continued use of a rubber stamp bearing the words "non specific", since all tissue samples reflect their specific physiological or pathological status when removed from their owner. Let's hope we will no longer see that rubber stamp uncritically wheeled out when, in the likely context of gluten sensitisation, a Marsh I or II lesion is present – but dismissed. It should likewise be realised that a flat Marsh III lesion is equally capable of being mimicked by an extensive list of other causes, and for that reason cannot be deemed "non-specific" either (See Table 2: (8)). Histopathologists only require lists of differential diagnoses for each phase (MO-3). And if they can remember those lists, then surely they could retain the four simple evolutionary steps through which the process of mucosal change was characterised.

 In continuing, I suspect that the Marsh I lesion (6) may persist in some individuals, possibly for a long time. Interestingly, we do not know whether it regresses, like the lymphocytic infiltration of the islets of some non-obese diabetic (NOD) female mice. The other possibility is that given the appropriate molecular signals (of which again we have no details) the mucosa begins moving away from Marsh I, passing rapidly through Marsh II, and thence onwards towards progressive villous effacement. That would be an interesting research exercise to elucidate.

 That the earliest gluten-induced lesions may be associated with some severe clinical symptoms and deficiencies now becomes highly relevant (10, 11), and that is another reason why diagnosis is made at the bedside. It should soberly remind us - contrary to Srivastava's view (12) that anything goes with his somewhat jokey *'much ado about nothing' *- that early lesions are of extreme importance clinically, and particularly with Marsh O. They should be given their due regard. As Pena remarked in quoting me (13), *'genes and gluten ingestion are logically anterior to the initiation of detectable mucosal abnormalities'*. And it is at this critical frontier in the encounter of gluten with the predisposing genes that immune and inflammatory cascades are recruited and deployed within the intestinal mucosa.

 Moreover, it is at that crucial interface where diagnostic potentiality lies at its most fragile. This dilemma has been brought into even more critical focus by the recent pandemic of cases of so-called "non-coeliac gluten sensitivity" – whatever those words supposedly could mean. Indeed, these circumstances have revealed the weaknesses in the recognition and diagnosis of gluten sensitivity when the condition is in its early phases of evolution, and when structural and immune markers (EMA; tTG-IgA) may not be up and running. In such cases, more specialised laboratory help is required, including HLA typing, staining for γδ IEL, culturing biopsies for tTG secretion, or looking for tTG-IgA deposits in subepithelial and microvascular basement membranes. 

 Elsewhere, I complained that a great deal of sophisticated immunological input is given to the Marsh III lesion. That's fine, but if all that type of work could be extended to each Marsh lesion from O through II, we would accrue a very comprehensive concept of what specific genes and their products are being recruited, and de-recruited, as the sequence advances. Seen from that perspective, we still have a very long way to go.

 So, lastly, to some theoretical points. It is particularly amusing for me to notice how the word "morphometry" comes to be used so disparagingly. When I came away from Boston, I was perturbed by the Ferguson technique (IEL "counts" per 100 enterocyte nuclei) as well as by the use of "high-power fields" in comparing cellular content between flat and normal mucosae. Using computerised image analysis technology as available then, I decided to employ an invariant (100µM^2^) test area of muscularis mucosae upon which to reconstruct each mucosal biopsy which was being processed in my lab. And indeed, in developing that technique further, Ensari subsequently, and magically, got immuno-stained, precise vertically-cut frozen sections into the computer for her morphometrically-based diagnostic study of rectal gluten challenge (14). Indeed, the use of this procedure could be usefully adopted in helping resolve the difficult distinction between early coeliacs, and the wheat intolerant group.

 A representative overview of a large number of mucosae analysed by our computerised methodology (Fig 7, p. 136 in (15)) reveals how much information can be depicted at a glance, but which would be totally impossible with only histological slides or microphotographs. There it is revealed how rapid changes in villi, crypts and lamina propria swelling begin with the Marsh II lesion, again pointing to its apparent *pivotal *location in the spectral continuum of mucosal change. Thus it is important that, even in diagnostic work, we do not lose sight of that important pivotal junction, *pace* Villanacci. And that raises further crucial questions, such as why Marsh I crypts are not infiltrated, and why lamina propria inflammation and inflammatory cell infiltrates only become apparent during the Marsh II phase. Also, is the *doubling* of crypt volumes (Marsh II lesions) due solely to T cell-mediated actions (16) and what factor(s) further increase crypt volumes *fourfold* in the Marsh III lesion, and with it the late, enormous outburst of crypt cell mitotic activity? And why are the crypts generally so unscathed, relative to the surface epithelium? And so on ......

 More importantly, these detailed morphometric studies embarrassingly revealed just how inaccurate the Ferguson technique is (17, 18, 19), since it over-estimates IEL numbers by up to a factor of two, due to the loss of nearly 50% enterocyte *nuclei* in sectioned tissue. (see Fig 12, in Ref 15 for an explanation as to why these nuclei are never seen, and thus lost to counting). Little wonder that there has been arbitrary agreement that the "upper limit of normal" is <25 IEL %. The problem here is that no empirically measurable cut-off point has ever been determined, since the IEL populations in control and coeliac mucosae are not bimodally distributed, but part of a continuum – rather like blood pressure, body weight, acid secretion.

 Furthermore, although most papers announce that the coeliac epithelium is "infiltrated" by IEL: that is probably incorrect. Indeed, as flattening occurs, the total IEL population falls dramatically, but to a lesser degree from that affecting the cells along the surface epithelium, which gives rise to this spurious notion when assessed by the erroneous Ferguson counting procedure. I could never understand (nor others (17)) the rationale of using one moving target in order to fix the movements of another! 

 This, I think, is another telling example of failure to notice relevant articles in the literature – that is, why the paper by Guix (17) has neither been recognised nor referred to, or indeed their counting grid ever used, in order to obtain more accurate counts. The intestinal mucosa is a complex piece of tissue (20), and cannot be reductively narrowed to accommodate some of the impoverished understandings that seemingly prevail these days.

 Finally, the remodelling of the coeliac mucosa, together with effacement of villi, is NOT – COULD NOT BE – due to "atrophic" process: if that were the case, then there could be no chance whatsoever of some observable regeneration taking place following dietary exclusion. Surely we have not already forgotten that mucosal regeneration was, for many years, taken as a late, but final, diagnostic criterion of gluten sensitivity. Indeed, if we refer back to Figure 7 (15), it becomes apparent that after Marsh II, the mucosa swings from a vertically-oriented structure to a pronounced *hypertrophic*, horizontally-based mucosal configuration, during which there is considerable swelling of the lamina propria - due to oedema as well as intense cellular infiltration. 

 That momentous change requires extensive cross-talk between epithelium and the mesenchyme constituting the lamina and its microvasculature, in order to co-ordinately bring about those striking influences operative in mucosal re-modelling. Again, we seem to have little perception as to how all this comes about. Some decent cell biology would help here. But what should be evident is that there are no "atrophic" or "partially degenerate" villi hanging on desperately during these final stages of remodelling, as Oberhuber struggles to maintain. Our paper demolished that archaic view (2). 

 We need far more understanding of the process of mosaic plateaux formation, including the microvascular alterations which permit these developments, and as the means whereby mucosal effacement is continually effected. Those old observations of surface mosaic plateaux - which seemingly are conglomerations of small groups of adjacent villi undergoing progressive and collective flattening - now seem to have been lost in translation, as they say.

 Clearly, there is much still to be learned in the future – as well as re-remembered from the past. 
